# Coronary Artery Disease: A Study on the Joint Role of Birth Weight, Adenosine Deaminase, and Gender

**DOI:** 10.4061/2009/860328

**Published:** 2010-04-13

**Authors:** F. Gloria-Bottini, M. Banci, P. Saccucci, N. Lucarini, F. Ianniello, G. Paradisi, A. Magrini, E. Bottini

**Affiliations:** ^1^Department of Biopathology and Imaging Diagnostics, University of Tor Vergata, 00133 Rome, Italy; ^2^Department of Cardiology, Valmontone Hospital, Rome, Italy; ^3^Department of Biology, MCA University of Camerino, Camerino, Italy; ^4^Unit of Gynecology, Valmontone Hospital, Rome, Italy; ^5^Department of Obstetrics and Gynaecology, Holy Hearth Catholic University, Rome, Italy

## Abstract

An inverse relationship between birth weight and coronary artery diseases is well documented but it remains unclear which exposure in early life might underlie such association. Recently it has been reported an association between adenosine deaminase genetic polymorphism and coronary artery diseases. Gender differences in the degree of this association have been also observed. These observations prompted us to study the possible joint effects of BW, ADA, and gender on the susceptibility to coronary artery diseases. 222 subjects admitted to hospital for nonfatal coronary artery diseases, and 762 healthy consecutive newborns were studied. ADA genotypes were determined by DNA analysis. A highly significant complex relationship has emerged among ADA, birth weight, and gender concerning their role on susceptibility to coronary artery diseases in adult life. Odds ratio analysis suggests that low birth weight is more important in females than in males. ADA^*∗*^2 allele appears protective in males, while in females such effect is obscured by birth weight.

## 1. Introduction

Recently an association between Adenosine Deaminase (ADA) genetic polymorphism and Coronary Artery Disease (CAD) has been reported [1]; subjects carrying the ADA*2 allele are less represented among CAD patients than in controls. We have confirmed in males this association [2]. An inverse relationship between birth weigh (BW) and CAD is well documented, and we have confirmed this association in females [3]. Since it remains unclear which exposure in early life might underlie the relationship between BW and CAD [4], in the present note we have reanalyzed our data to search for possible interactions among BW, ADA, and gender concerning susceptibility to CAD.

### 1.1. Adenosine Deaminase Genetic Polymorphism

ADA is a polymorphic enzyme present in all mammalian tissues [5]. It is controlled by a locus with 2 codominant alleles ADA*1 and ADA*2 located on the long arm of chromosome 20 [6]. ADA catalyses the irreversible deamination of adenosine to inosine. Red blood cells (RBCs) are in equilibrium with freely diffusing adenosine [7] pointing to an important role for this enzyme in the regulation of adenosine concentration.

Adenosine is an important local hormone regulating blood flow, neurotransmission, physiology of smooth muscle, and platelet aggregation. In the liver adenosine counteracts insulin action by activating A2B receptors [8].

ADA and CD26 are colocalized on T-cell surface, but not inside cells. Cells expressing ADA and CD26 on the surface are much more resistant to the inhibitory effects of adenosine.

Through the regulation of glucose metabolism, genetic variation of adenosine concentration due to ADA polymorphism may have an important role in intrauterine development and bilirubin metabolism in the newborn.

Severe ADA deficiency is a systemic metabolism disorder associated to organ dysfunction [9]. The possible metabolic and clinical effects of the common ADA*2 variant that is associated with a 15% reduction of enzymatic activity have not received much attention in the medical literature but it is likely that it could be responsible for variation of metabolic and clinical parameters.

## 2. Material and Methods

222 subjects admitted consecutively to the Hospital for non fatal CAD were studied. 762 consecutive newborn infants were considered as controls. All subjects were Italians from the White population of Central Italy.

ADA phenotype/genotype was determined by DNA analysis in CAD subjects [10] and by starch gel electrophoresis [5] in newborns. In our laboratory no difference has been observed between the two methods. Moreover in the population of the Central Italy we have observed the same distribution of ADA genotypes (phenotypes) by the two methods.

Reliable information on Birth Weight was obtained in 126 subjects with CAD.

Chi-square test of independence, correlation analysis, and principal component analysis were carried out by SPSS package [11]. Principal Component Analysis gives a synthesis of the overall pattern of correlations among variables. Starting from a correlation matrix, the method extracts independent components giving the contribute of original variables to each component. A component showing an Eigenvalue greater than 1 is considered significant. Three-way contingency table analysis was carried out by a log-linear model according to Sokal and Rohlf [12]. Clinical data in subjects with CAD have been reported in a previous paper [3].

## 3. Results

Table 1 shows the pattern of the correlations among BW, ADA, and gender. The correlation between ADA and gender in CAD patients is opposite to that in healthy newborns. The correlation between gender and BW is highly significant in CAD only and the correlation between ADA and BW is border line in CAD and is absent in newborns.

Table 2 shows the results of Principal Component Analysis performed on BW, ADA, and gender. There are important difference in all parameters considered between CAD and newborns suggesting a peculiar pattern of correlation among BW, ADA, and gender in CAD. This is particularly evident considering the contribution of the three variables to the first component.

In the following tables the pattern of correlations among the three variables has been analyzed in details.

Table 3 shows the distribution of ADA phenotypes in healthy newborns and in CAD patients. In males, the proportion of carriers of ADA*2 allele is significantly lower in CAD than in controls. In females the difference is not statistically significant. A three way contingency table analysis by a log-linear model suggests that the association of ADA with CAD depends on gender.

Table 4 shows the relationship between BW and gender in healthy newborns and in CAD patients. We have considered two classes: BW ≤ 3000 g (corresponding to the first quartile) and BW > 3000 g. In CAD the proportion of subjects with a BW ≤ 3000 g is much greater in females than in males. This is not observed among healthy newborns.

Table 5 shows the distribution of ADA phenotypes in relation to gender in healthy newborns and in CAD separately for subjects with a BW ≤ 3000 g and for those with a BW > 3000 g. The interaction among ADA phenotype, gender, and disease observed in Table 3 is statistically significant in subjects with a low BW only suggesting a complex interaction among ADA, gender, and BW concerning their effects on susceptibility to CAD.

Table 6 shows the distribution of BW in relation to ADA and gender in healthy newborns and in CAD. In CAD, ADA and BW contribute significantly to differentiate males from females. No significant effect of these variables is observed in healthy newborns.

In Table 7 we have summarized the complex relationship between ADA, BW, gender and susceptibility to CAD. A highly significant interaction is observed. In females the joint ADA-BW phenotype distribution shows a highly significant difference between CAD and newborns while in males no significant association is observed. Concerning susceptibility to CAD Odds ratio analysis suggests that low BW is more important in females than in males. ADA*2 allele appears protective in males, while in females such effect is obscured by the effect of BW.

## 4. Discussion

Correlation analysis shows that the pattern of correlations among BW, gender, and ADA in subject with CAD is significantly different from that observed in healthy subjects. ADA*2 allele is less represented in CAD males as compared to females but such difference is evident in subjects with a relatively low birth weight only. On the other hand a direct association between BW, and CAD is observed in females only. Moreover CAD females carrying the ADA*2 allele shows a lower BW compared to ADA*1/*1 females but such association is not observed in males. Overall, this indicates a complex interaction involving gender, BW and ADA concerning their effects on susceptibility to CAD. Statistical analysis suggests that in males prevails the effect of ADA polymorphism, while in females prevails the effect of BW.

The lower activity of ADA*2 allele compared to ADA*1 results in higher concentration of adenosine that in turn may exert a positive role on cardiac function. This may represent a possible explanation of the negative association between ADA*2 allele and CAD. Indeed ecto-ADA interacts with Adenosine Receptors A_1_ that influences ischemic preconditioning [13–16]. Moreover adenosine influences platelet aggregation [16] and has important immunomodulatory property [17].

The developmental origin of adult diseases theory postulates that foetal undernutrition increases the susceptibility to coronary artery disease and allied disorders in later stages of life [18]. The inverse association observed between BW and CAD supports this hypothesis. Gender difference in the pattern of association between BW and CAD has been also reported [4]. However it remains to discover the factors acting in early life that might be responsible for the association between development and susceptibility to an important class of adult diseases [4]. Studies on adults exposed to maternal undernutrition have not demonstrated an association with CAD [19] suggesting that other unknown factors, probably also genetic, could have an important role in the link between development and susceptibility to CAD in adult life.

In this context based on the association of ADA polymorphism with peculiar neonatal events we have proposed another line of investigation [20].

We have shown that ADA genetic variability influences neonatal bilirubin levels. In particular ADA*2 allele is more represent in newborns with high serum bilirubin level as compared to newborns with low level [21, 22]. On the other hand several studies indicate a beneficial action of bilirubin in the neonatal period due to its protective effects from secondary oxidant formed in the oxidative process [23–25].

We have observed that the proportion of carriers of ADA*2 allele in newborns who have undergone phototherapy for high bilirubin level during the first few days of life is higher as compared to those who have not undergone such treatment [21, 22]. On the other hand the proportion of carriers of ADA*2 allele is lower in CAD than in healthy subjects [1]. The pattern is suggestive; the association between ADA and CAD might have origin from peculiar events occurring in early stages of extra uterine life.

Since the newborn is particularly susceptible to oxidative damage, the effect of ADA*2 allele on bilirubin level could result in a protection against oxidative damage and in turn in long-term positive effect on the protection towards CAD in adult life. Hormonal diversity could be responsible for gender differences. These differences, however, could be the expression of different clinical severity leading to different rates of mortality between sexes. Indeed our cases include nonfatal CAD only, and we have no information on fatal cases.

## 5. Study Limitations

The present observations should be confirmed in another clinical setting before drawing definitive conclusion. An important limitation is due to lack of case fatality rate that might be different between sexes influencing the degree of correlations observed in our cases.

## Figures and Tables

**Table 1:** Correlation between variables in subjects with CAD and in newborn infants from the same population.

	CAD		Healthy newborns	
Correlation	Coefficient	Significance (*P*)	Coefficient	Significance (*P*)
Gender-ADA	−0.159	.016	0.087	.092
ADA-BW	−0.165	.066	−0.038	.461
Gender-BW	0.351	.000	0.002	.971

**Table 2:** Principal component analysis.

		CAD		Healthy newborns	
Communalities	Sex	0.598		0.449	
	BW	0.552		0.110	
	ADA	0.338		0.537	

		Eigenvalue	%variance	Eigenvalue	%variance

Total variance explained	1° component	1.487	49.56%	1.096	36.53%
	2° component	0.861	28.70%	0.997	33.22%
	3° component	0.652	21.74%	0.908	30.25%

		1rst component		1rst component	

Component matrix	Sex	0.773		0.670	
	BW	0.743		−0.331	
	ADA	−0.581		0.733	

**Table 3:** Distribution of ADA phenotypes in healthy newborns and in subjects with CAD.

	Healthy newborns	CAD	Chi square test of independence		
			*χ* ^2^	df	*P*
*Females*					
% of carriers of ADA*2 allele	15.7%	18.3%	0.3606	1	.620
total no.	368	104			
*Males*					
% of carriers of ADA*2 allele	15.5%	7.6%	4.637	1	.035
total no.	394	118			

Three-way contingency table analysis by a log-linear model:			
x = samples (healthy newborn versus CAD) y = ADA, and z = gender.			
x y z interaction:	G	df	*P*
	4.382	1	.042

**Table 4:** Birth weight in relation to gender in healthy newborns and in subjects with CAD.

	Healthy newborns	CAD	Chi square test of independence		
			^2^ *χ*	df	*P*
*Females*					
% proportion of newborns with a BW ≤ 3000 g*	25.8%	59.3%	26.677	1	<10^−6^
total no.	368	59			
*Males*					
% proportion of newborns with a BW ≤ 3000 g*	22.1%	25.4%	0.105	1	.745
total no.	394	67			

*3000 g corresponds to the first quartile.

**Table 5:** Distribution of ADA phenotypes in relation to gender and birth weight in healthy newborns and in subjects with CAD.

BW ≤ 3000 g	Healthy newborns	CAD
*Females*		
% of carriers of ADA*2 allele	13.7%	28.6%
total no.	95	35
*Males*		
% of carriers of ADA*2 allele	17.2%	5.9%
total no.	87	17

Three-way contingency table analysis by a log-linear model		
x = samples (healthy newborn vs CAD), y = ADA, and z = gender		
x y z interaction G	df	*P*
4.495	1	.037

BW > 3000 g		

*Females*		
% of carriers of ADA*2 allele	16.5%	12.5%
total no.	273	23
*Males*		
% of carriers of ADA*2 allele	15.0%	8.0%
total no.	307	50

Three-way contingency table analysis by a log-linear model		
x = samples, y = ADA, and z = gender		
x y z interaction G	df	*P*
0.205	1	.680

**Table 6:** Birth weight in relation to ADA phenotype in healthy newborn and in subjects with CAD.

	Healthy newborns		CAD	
	ADA 1/1	Carriers of ADA*2 allele	ADA 1/1	Carriers of ADA*2 allele
*Females*				
% proportion of newborns with a BW ≤ 3000 g	26.5%	22.4%	54.3%	76.9%
total no.	310	58	46	13
*Males*				
% proportion of newborns with a BW ≤ 3000 g	21.6%	24.6%	25.8%	20.0%
total no.	333	61	62	5

Three-way contingency table analysis by a log-linear model x = ADA, y = BW, and z = gender						
x y z interaction	G	df	*P*	G	df	*P*
	0,674	1	.430	1	1.121	.320
Independence of factor z from factors x and y	2.145	3	.380	19.732	3	.0002

**Table 7:** The relationship among joint ADA-BW phenotype, gender, and sample (CAD versus newborn).

		CAD		Newborns	
		Females	Males	Females	Males
	ADA1/1 BW ≤ 3000 g (A)	42.2%	23.9%	22.3%	18.3%
Joint ADA-BW phenotype	ADA1/1 BW > 3000 g (B)	35.6%	68.7%	62.0%	66.2%
	ADA*2 carrier BW ≤ 3000 g (C)	16.9%	1.5%	3.5%	3.8%
	ADA*2 carrier BW > 3000 g (D)	5.1%	6.0%	12.2%	11.7%
	total no.	59	67	368	394

Three-way contingency table analysis by a log-linear model			
x = gender, y = joint ADA-BWphenotype, and z = sample (CAD versus newborns)			
x y z interaction	G	df	*P*
	13.605	3	.005

Chi-square test of independence (CAD versus newborns)			
	*χ* ^2^	df	*P*
Female/	33.551	3	<10^−6^
Males	3.595	3	.308

Odds ratio analysis (CAD versus newborns)					
		Joint ADA-BW phenotype			
		A versus others	B versus others	C versus others	D versus others
Females	OR	2.564	0.339	5.573	0.384
	95% CI	1.392–4.716	0.183–0.623	2.130–14.487	0.099–1.346
Males	OR	1.403	1.116	0.382	0.480
	95% CI	0.721–2.701	0.619–2.024	0.016–2.83	0.141–1.457

## References

[1] Safranow K, Rzeuski R, Binczak-Kuleta A (2007). ADA^*∗*^2 allele of the adenosine deaminase gene may protect against coronary artery
disease. *Cardiology*.

[2] Banci M, Saccucci P, D’Annibale F (2009). Adenosine deaminase genetic polymorphism and coronary artery disease. *Cardiology*.

[3] Banci M, Saccucci P, Dofcaci A (2009). Birth weight and coronary artery disease. The effect of gender and diabetes. *International Journal of Biological Sciences*.

[4] Huxley R, Owen CG, Whincup PH (2007). Is birth weight a risk factor for ischemic heart desease in later life?. *American Journal of Clinical Nutrition*.

[5] Spencer N, Hopkinson D, Harris H (1968). Adenosine deaminase polymorphism in man. *Annals of Human Genetics*.

[6] Honig J, Martiniuk F, D’Eustachio P (1984). Confirmation of the regional localization of the genes for human acid alpha-glucosidase (GAA) and adenosine deaminase (ADA) by somatic cell hybridization. *Annals of Human Genetics*.

[7] McKusik VA (1994). *Mendelian Inheritance in Man*.

[8] Yasuda N, Inoue T, Horizoe T (2003). Functional characterization of the adenosine receptor contributing to glycogenolysis and gluconeogenesis in rat hepatocytes. *European Journal of Pharmacology*.

[9] Bollinger ME, Arredondo-Vega FX, Santisteban I, Schwarz K, Hershfield MS, Lederman HM (1996). Hepatic dysfunction as a complication of adenosine deaminase deficiency. *The New England Journal of Medicine*.

[10] Bottini N, De Luca D, Saccucci P (2001). Autism: evidence of association with adenosine deaminase genetic polymorphism. *Neurogenetics*.

[12] Sokal RR, Rohlf FJ (1981). *Biometry*.

[13] Ciruela F, Saura C, Canela EI, Mallol J, Lluis C, Franco R (1996). Adenosine deaminase affects ligand-induced signalling by interacting with cell surface adenosine receptors. *FEBS Letters*.

[14] Heusch G, Schulz R (1997). Endogenous protective mechanisms in myocardial ischemia: hibernation and ischaemic preconditioning. *American Journal of Cardiology*.

[15] Lankford AR, Yang JN, Rose’Meyer R (2006). Effect of modulating cardiac A1 adenosine receptor expression on protection with ischaemic preconditioning. *American Journal of Physiology*.

[16] Hayes ES (2003). Adenosine receptors and cardiovascular disease: the adenosine-1 receptor (A1) and A1 selective ligands. *Cardiovascular Toxicology*.

[17] McCallion K, Harkin DW, Gardiner KR (2004). Role of adenosine in immunomodulation: review of the literature. *Critical Care Medicine*.

[18] Barker DJP (1993). The intrauterine origins of cardiovascular disease. *Acta Paediatrica, Supplement*.

[19] Kannisto V, Christensen K, Vaupel JW (1997). No increased mortality in later life for cohorts born during famine. *American Journal of Epidemiology*.

[20] Bottini E, Gloria-Bottini F (2009). Genetic polymorphism of adenosine deaminase and early neonatal events: is there an association with susceptibility to coronary artery disease in adult life?. *Cardiology*.

[21] Lepore A, Lucarini N, Evangelista MA (1989). Enzyme variability and neonatal jaundice. The role of adenosine deaminase and acid phosphatase. *Journal of Perinatal Medicine*.

[22] Gloria-Bottini F, Magrini A, Cozzoli E, Bergamaschi A, Bottini E (2008). ADA genetic polymorphism and the effect of smoking on neonatal bilirubinemia and developmental parameters. *Early Human Development*.

[23] Bélanger S, Lavoie J-C, Chessex P (1997). Influence of bilirubin on the antioxidant capacity of plasma in newborn infants. *Biology of the Neonate*.

[24] Dailly E, Urien S, Barré J, Reinert P, Tillement JP (1998). Role of bilirubin in the regulation of the total peroxyl radical trapping antioxidant activity of plasma in sickle cell disease. *Biochemical and Biophysical Research Communications*.

[25] Minetti M, Mallozzi C, Di Stasi AMM, Pietraforte D (1998). Bilirubin is an effective antioxidant of peroxynitrite-mediated protein oxidation in human blood plasma. *Archives of Biochemistry and Biophysics*.

